# Classification of the Disposition of Patients Hospitalized with COVID-19: Reading Discharge Summaries Using Natural Language Processing

**DOI:** 10.2196/25457

**Published:** 2021-02-10

**Authors:** Marta Fernandes, Haoqi Sun, Aayushee Jain, Haitham S Alabsi, Laura N Brenner, Elissa Ye, Wendong Ge, Sarah I Collens, Michael J Leone, Sudeshna Das, Gregory K Robbins, Shibani S Mukerji, M Brandon Westover

**Affiliations:** 1 Department of Neurology Massachusetts General Hospital Boston, MA United States; 2 Clinical Data Animation Center Boston, MA United States; 3 Harvard Medical School Boston, MA United States; 4 Division of Pulmonary and Critical Care Medicine Massachusetts General Hospital Boston, MA United States; 5 Division of General Internal Medicine Massachusetts General Hospital Boston, MA United States; 6 Division of Infectious Diseases Massachusetts General Hospital Boston, MA United States; 7 McCance Center for Brain Health Massachusetts General Hospital Boston, MA United States

**Keywords:** ICU, coronavirus, electronic health record, unstructured text, natural language processing, BoW, LASSO, feature selection, machine learning, intensive care unit, COVID-19, EHR

## Abstract

**Background:**

Medical notes are a rich source of patient data; however, the nature of unstructured text has largely precluded the use of these data for large retrospective analyses. Transforming clinical text into structured data can enable large-scale research studies with electronic health records (EHR) data. Natural language processing (NLP) can be used for text information retrieval, reducing the need for labor-intensive chart review. Here we present an application of NLP to large-scale analysis of medical records at 2 large hospitals for patients hospitalized with COVID-19.

**Objective:**

Our study goal was to develop an NLP pipeline to classify the discharge disposition (home, inpatient rehabilitation, skilled nursing inpatient facility [SNIF], and death) of patients hospitalized with COVID-19 based on hospital discharge summary notes.

**Methods:**

Text mining and feature engineering were applied to unstructured text from hospital discharge summaries. The study included patients with COVID-19 discharged from 2 hospitals in the Boston, Massachusetts area (Massachusetts General Hospital and Brigham and Women’s Hospital) between March 10, 2020, and June 30, 2020. The data were divided into a training set (70%) and hold-out test set (30%). Discharge summaries were represented as bags-of-words consisting of single words (unigrams), bigrams, and trigrams. The number of features was reduced during training by excluding n-grams that occurred in fewer than 10% of discharge summaries, and further reduced using least absolute shrinkage and selection operator (LASSO) regularization while training a multiclass logistic regression model. Model performance was evaluated using the hold-out test set.

**Results:**

The study cohort included 1737 adult patients (median age 61 [SD 18] years; 55% men; 45% White and 16% Black; 14% nonsurvivors and 61% discharged home). The model selected 179 from a vocabulary of 1056 engineered features, consisting of combinations of unigrams, bigrams, and trigrams. The top features contributing most to the classification by the model (for each outcome) were the following: “appointments specialty,” “home health,” and “home care” (home); “intubate” and “ARDS” (inpatient rehabilitation); “service” (SNIF); “brief assessment” and “covid” (death). The model achieved a micro-average area under the receiver operating characteristic curve value of 0.98 (95% CI 0.97-0.98) and average precision of 0.81 (95% CI 0.75-0.84) in the testing set for prediction of discharge disposition.

**Conclusions:**

A supervised learning–based NLP approach is able to classify the discharge disposition of patients hospitalized with COVID-19. This approach has the potential to accelerate and increase the scale of research on patients’ discharge disposition that is possible with EHR data.

## Introduction

The COVID-19 pandemic continues to present challenges for health care systems around the world [1-8], with over 32.7 million COVID-19 cases confirmed and 991,000 deaths worldwide as of September 27, 2020 [6]. The SARS-CoV-2 virus first appeared in Wuhan, China, in December 2019. The first case in the United States was confirmed January 20, 2020 [9], followed by rapid spread [2]. By the end of April, Massachusetts became the third hardest hit state, trailing New York and New Jersey [10].

To prepare for a possible second wave in Massachusetts, we set out to conduct a large-scale study of factors associated with outcomes in hospitalized patients at 2 large academic Boston hospitals. This effort required the significant task of reviewing medical records for over 1000 patients. For structured parts of the electronic health record (EHR), automated data extraction is straightforward. However, some essential information is exclusively or most reliably available only in semistructured or unstructured narrative medical notes, including patient-reported symptoms, examination findings, or social habits. Thus, developing automated approaches to EHR information extraction wherever possible is critical for more complete patient phenotyping.

Natural language processing (NLP) deals with automated analysis of unstructured text data. Recent advances in NLP machine learning have empowered computers to do several tasks such as machine translation, speech recognition, speech synthesis, semantic understanding, and text summarization [11,12]. NLP has the advantage of being much faster than human chart review of medical records [13-16].

Here we present an automated approach, using NLP, to extract a specific outcome from hospital discharge summaries: discharge destination or “disposition” (ie, anticipated location or status following discharge). Dispositions of interest included home, inpatient rehabilitation center, skilled nursing inpatient facility (SNIF), and death. Discharge disposition of patients with COVID-19 from health care facilities is important due to the high risk of transmission of the disease within nursing homes and hospitals when patients are discharged to locations other than home, and also because it represents an important measure closely related to functional outcome and level of disability following hospitalization, as well as overall costs of care. Furthermore, this information has the potential to aid health care facilities in resource planning to better prepare for the incoming flow of patients. Although our model is tailored for discharge disposition, the approach we developed is generalizable to other outcomes available in discharge summaries.

## Methods

### Study Overview

Data were extracted from the hospital electronic medical record under a research protocol approved for a waiver of informed consent by the Partners Healthcare Institutional Review Board. Clinical data were retrospectively analyzed for all adult patients who tested positive for SARS-CoV-2 infection between March 10 and June 30, 2020. A total of 1737 patients admitted to 2 major Boston hospitals, 1232 from Massachusetts General Hospital (MGH) and 505 from Brigham and Women’s Hospital (BWH), were included. Only patients with a physician discharge summary and available known ground-truth discharge disposition were included.

### Data Collection and Processing

#### Overview

**Figure 1:**
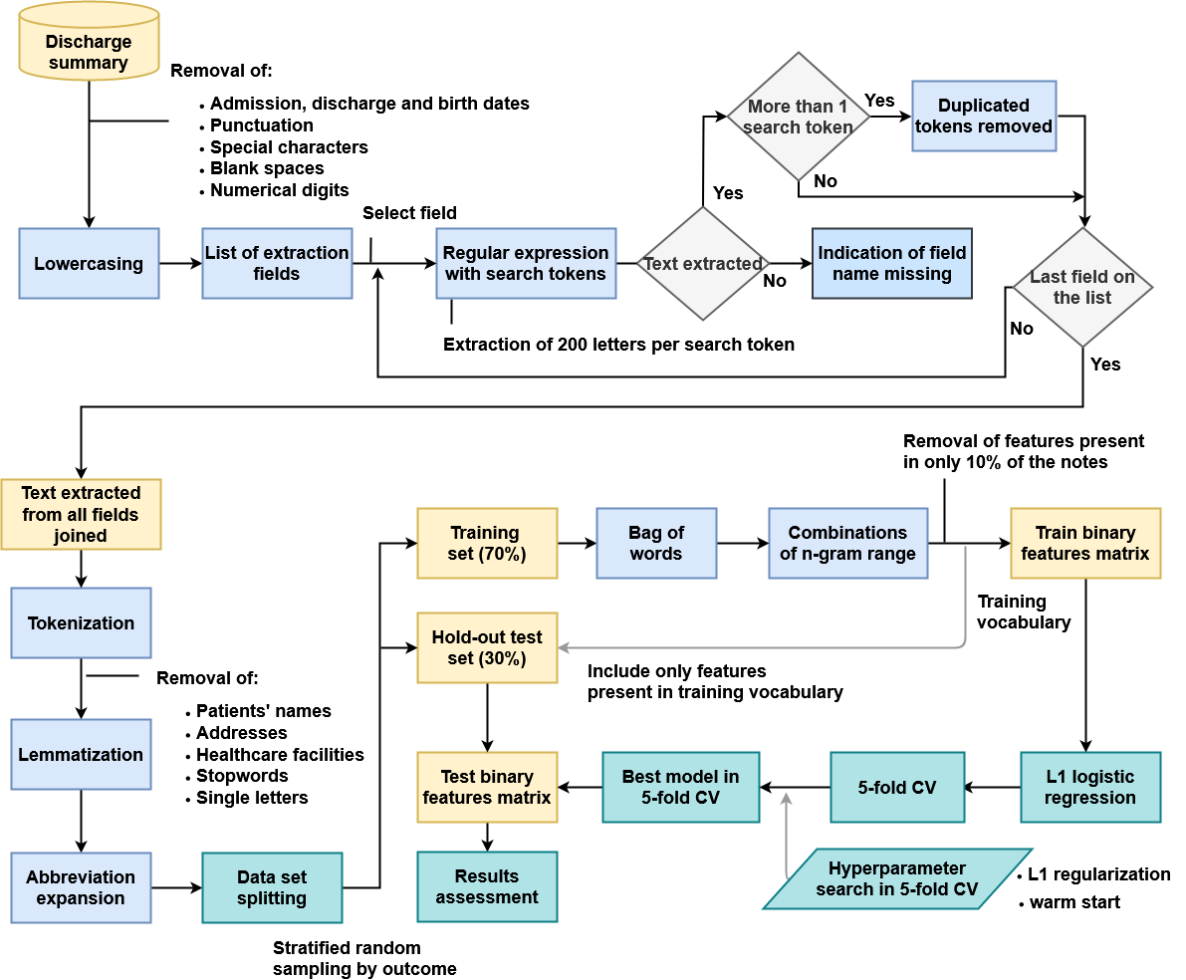
Methodology steps for discharge summary notes preprocessing and modeling. The list of extraction field is depicted in Table 1.

Data consisted of discharge summaries, which are unstructured free-text notes written by physicians, and a ground-truth record of discharge disposition, used to assess the accuracy of the NLP results. The methodology for note preprocessing is shown in Figure 1. The upper part of the figure provides an overview of the text extraction for each field on the list of extraction fields depicted in Table 1. The lower part of the figure shows the methodology steps where the text extracted from all the fields is processed for modeling. The data were randomly stratified into train and test sets for modeling, which we address in the Model Development section.

**Table 1:** Information captured from discharge summaries, grouped in fields, and respective search tokens used in the regular expression.

Field	Search token
Discharge disposition	“discharge,” “discharged,” “dispo,” “skilled nursing,” “snf”
Diagnosis	“diagnosis,” “diagnoses,” “problem,” “reason for admission,” “chief complaint”
Surgeries	“surgeries this admission”
Treatments	“treatments”
Tests	“tests”
Allergies	“allergies,” “allergic”
Diet	“diet,” “nutrition”
Medical history	“history”
Hospital course	“hospital course”
Laboratory results	“labs”
Activity	“activity,” “activities”
Physical exam	“discharge exam,” “physical exam”
Physical therapy	“physical therapy”
Occupational therapy	“occupational therapy”
Discharge instructions	“instructions”
Follow-up care	“follow up”
Discharge plan	“discharge plan”
Additional orders	“additional orders”
Code status	“code status”

#### Document Preprocessing

Admission, discharge, and birth dates were removed from the discharge summaries, as well as punctuation, special characters, blank spaces, and numerical digits. Notes were then subjected to lowercasing, tokenization, and correction using lemmatization, a procedure for obtaining the root form of the word, using vocabulary (dictionary importance of words) and morphological (word structure and grammar relations) analysis. *WordNetLemmatizer* from NLTK library in Python (Version 3.7; Python Software Foundation) was used with a part-of-speech (POS) tag specified as a verb. Patients’ names, addresses, health care facilities, and hospital unit names were removed, as well as single letters. Abbreviation expansion and spelling corrections were performed for a small list of frequently used clinical words (Table S1 in Multimedia Appendix 1). A list of commonly used and less informative stopwords was also removed from the notes (Table S2 in Multimedia Appendix 1).

#### Processing of Specific Discharge Summary Fields

Discharge summaries at MGH and BWH are semistructured, with a series of named fields containing specific types of mostly free-text information (Table 1). We present an example of discharge summary notes with protected health information removed (Table S3 in Multimedia Appendix 1). Text fields were identified based on information extracted from the notes using regular expressions with search tokens (Table 1). The function “str.extractall” from Python was used to extract a length of 200 letters of text onwards from all instances where the search token appeared.

Some notes contained a “discharge disposition” field used to list the discharge disposition. We deleted this field to avoid an overly “easy” solution, because this field is not universally available, and because we wished to assess how well the approach is able to perform when structured data is unavailable. In a field where more than one extraction was performed (ie, with more than one search token), the corresponding results were joined, and duplicated words were removed. To illustrate with an example, for the “Diet” field, using the regular expressions with search tokens “diet” and “nutrition,” 200 letters were captured for each search token, for a total of 400 letters. Since there might be repeated information in the discharge summary regarding diet and nutrition recommendations, duplicated words were removed from the captured text. Where no data was captured with the search tokens, an indication of missingness was set with the name of the field and the suffix “_missing.”

The texts extracted from all fields (depicted in Table 1) were joined to create a reduced version of the discharge summary, which was then subjected to tokenization, lemmatization, and abbreviation expansion, as described in the Document Preprocessing subsection. The vocabulary used for modeling was created based on these reduced versions of the discharge summaries contained in the training set. Documents were represented as a binary bag-of-words (BoW; ie, an ordered series of binary vectors indicating whether a given n-gram [word or sequence of 2 or 3 words] is present in the document, disregarding grammar and word order). The function *CountVectorizer* was used with its default parameters from Python, except for the n-gram range, which was set as unigrams (1 word), bigrams (2 consecutive words), and trigrams (3 consecutive words). As a first step to reduce dimensionality, only features present in at least 10% of the reduced version of the discharge summary notes were considered. Multiclass logistic regression with the least absolute shrinkage and selection operator (LASSO) [17] was used to further sparsify the model.

### Outcome Measure

The multiclass outcome measure was discharge disposition, composed of the classes: home, inpatient rehabilitation, SNIF, and death. “Home” included “home or self-care,” “home-health care services,” and patients who “left against medical advice.” SNIF included “Skilled Nursing Facility” and “Custodial Care Facility.”

### Model Development

The training algorithm used the one-vs-rest scheme for multiclassification, where a binary problem was fitted for each class and the class weight was balanced. Logistic regression [18] with LASSO regularization was used as the classification model. The model estimator 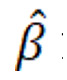 is depicted in equation 1 and the LASSO regularization objective can be written as in equation 2. 
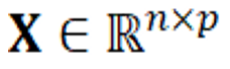
 corresponds to the design input matrix and 
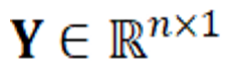
 corresponds to the vector of observations, where *n* is the number of observations, in this case the number of discharge summaries or number of patients, and *p* the number of features in 
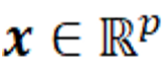
. The vector of regression coefficients is given by 
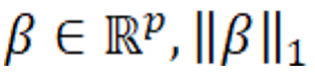
 corresponds to the L1 norm of this coefficients vector, and λ is the regularization parameter that controls the amount of shrinkage. The regularization adds a penalty on the weights to prevent overfitting [19]. The inverse of the regularization strength *C* was varied for the values {0.005, 0.01, 0.02, 0.03, 0.04, 0.05, 0.06, 0.07, 0.08, 0.09, 0.1, 0.5, 1, 1.5, 2, 2.5, 3, 3.5, 4, 4.5, 5}.


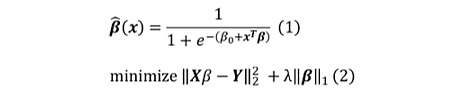


Stratified random sampling was used to split the data set into a training set (70%) and a hold-out test set (30%). A randomized search was used for hyperparameter tuning during training with 100 iterations of 5-fold cross-validation. The solver was set to “liblinear” and the “warm start” hyperparameter was varied between true/false, where “true” corresponded to reusing the solution of the previous call to fit as initialization, and “false” corresponded to erasing the previous solution.

### Performance Measures

The *R*^2^ coefficient of determination score was used in cross-validation scoring to select the best model configuration in the training data. The one standard error rule was used to select the regularization parameter. The simplest model, whose *R*^2^ mean score fell within 1 standard deviation of the maximum *R*^2^, was selected.

To measure model performance on test data, the area under the receiver operating characteristic curve (AUROC) was calculated. The ROC curve is a function of recall (sensitivity) versus the false positive rate (FPR; ie, 1–specificity; Table S1 in Multimedia Appendix 1). The pair (Recall_k_, FPR_k_) is called an operating point for this curve, where k is a threshold that is varied to generate the ROC curve. The equations for these metrics are presented in Table S4 in Multimedia Appendix 1.

The area under the precision-recall curve (AUPRC), which is an important measure in the presence of class imbalance, was also calculated. The pair (Recall_k_, Precision_k_) is referred to as an operating point for this curve. Average precision (AP; Table S3 in Multimedia Appendix 1) summarizes this plot as the weighted mean of precisions achieved at each threshold, with the increase in recall from the previous threshold used as the weight.

The F_1_-score (Table S4 in Multimedia Appendix 1) was also assessed as another performance metric commonly reported for data sets with imbalanced numbers across classes [20].

In total, 100 iterations of bootstrap random sampling with replacement were performed to calculate 95% CIs for performance metrics.

## Results

### Summary of Patient Population

From 1917 patients’ medical records, 1752 had a physician discharge summary and a discharge disposition within the categories of home, inpatient rehabilitation, SNIF, and death. Only adults (aged ≥18 years) were included in the analysis, leaving a study cohort of 1737 patients. The cohort was split into train and test sets using stratified random sampling according to outcome. Age in the train and test sets was balanced, with a median of 62 and 60 years old, respectively (Table 2). The majority of patients were White (n=774; median 44.6%) and Black or African American (n=285; median 16.4%). Most were discharged home (n=1052; 60.6%). Among all patients with COVID-19 in this sample, there were 243 (14.0%) nonsurvivors.

**Table 2:** Baseline characteristics of the study patient population stratified by train and test sets.

Characteristic		Train set (n=1215)	Test set (n=522)	Total (N=1737)
Age (years), median (SD)		62.0 (18.2)	60.0 (18.2)	61.0 (18.2)
**Gender, n (%)**				
	Female	545 (44.9)	244 (46.7)	789 (45.4)
	Male	670 (55.1)	278 (53.3)	948 (54.6)
**Race, n (%)**				
	White	533 (43.9)	241 (46.2)	774 (44.6)
	Hispanic or Latino	52 (4.2)	19 (3.6)	71 (4.1)
	Black or African American	204 (16.8)	81 (15.5)	285 (16.4)
	Asian	46 (3.8)	21 (4.0)	67 (3.9)
	American Indian or Alaska Native	31 (2.5)	13 (2.5)	44 (2.5)
	Native Hawaiian or other Pacific Islander	2 (0.2)	1 (0.2)	3 (0.2)
	Unknown^a^	347 (28.6)	146 (28.0)	493 (28.3)
**Institution, n (%)**				
	Massachusetts General Hospital	881 (72.5)	351 (67.2)	1232 (70.9)
	Brigham and Women’s Hospital	334 (27.5)	171 (32.8)	505 (29.1)
**Discharge disposition, n (%)**				
	Home	736 (60.6)	316 (60.5)	1052 (60.6)
	Inpatient rehabilitation	102 (8.4)	44 (8.4)	146 (8.4)
	Skilled nursing inpatient facility	207 (17.0)	89 (17.1)	296 (17.0)
	Death	170 (14.0)	73 (14.0)	243 (14.0)

^a^Unknown includes “other,” “declined,” or “unavailable.”

The preprocessed data set for modeling was created based on the notes extracted in all fields except the “discharge disposition” and “code status” fields, as described in the Processing of Specific Discharge Summary Fields subsection. Before dimensionality reduction, where features present in at least 10% of the reduced version of the discharge summary notes were considered, there were a total of 15,182 tokens (unigrams). After applying this dimensionality reduction step, we were left with 477 tokens. With this set of tokens, 3497 combinations of n-grams were generated, leading to a total of 1056 features with duplicates removed. Thus, the total number of candidate features in the training vocabulary was 1056, including 460 unigrams, 329 bigrams, and 267 trigrams.

### Modeling Results

**Figure 2:**
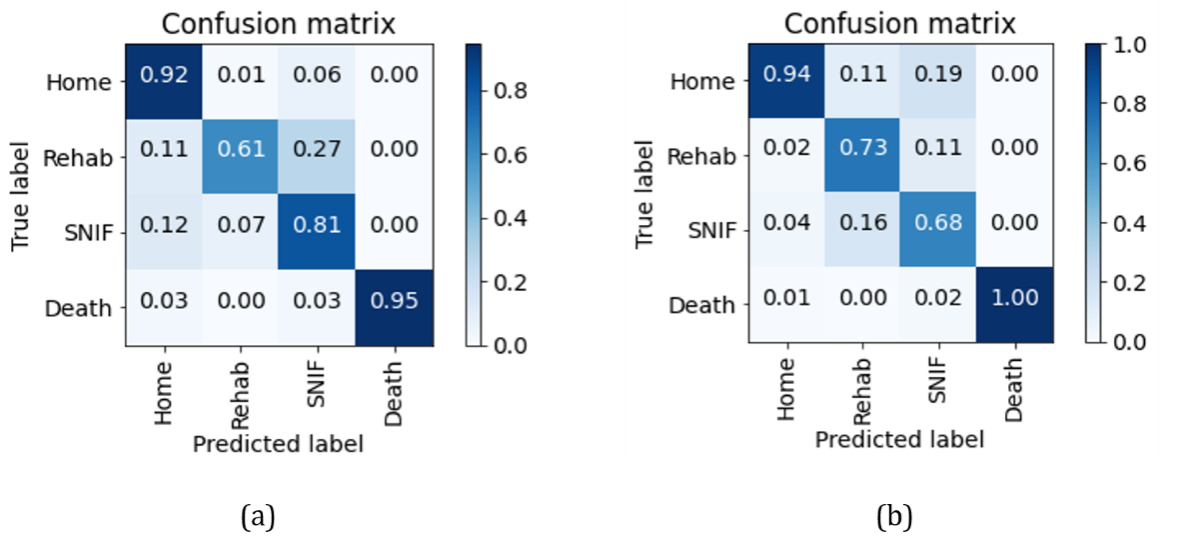
Confusion matrices for the best model evaluated in the hold-out test set normalized (A) by recall and (B) by precision. SNIF: skilled nursing inpatient facility.

**Figure 3:**
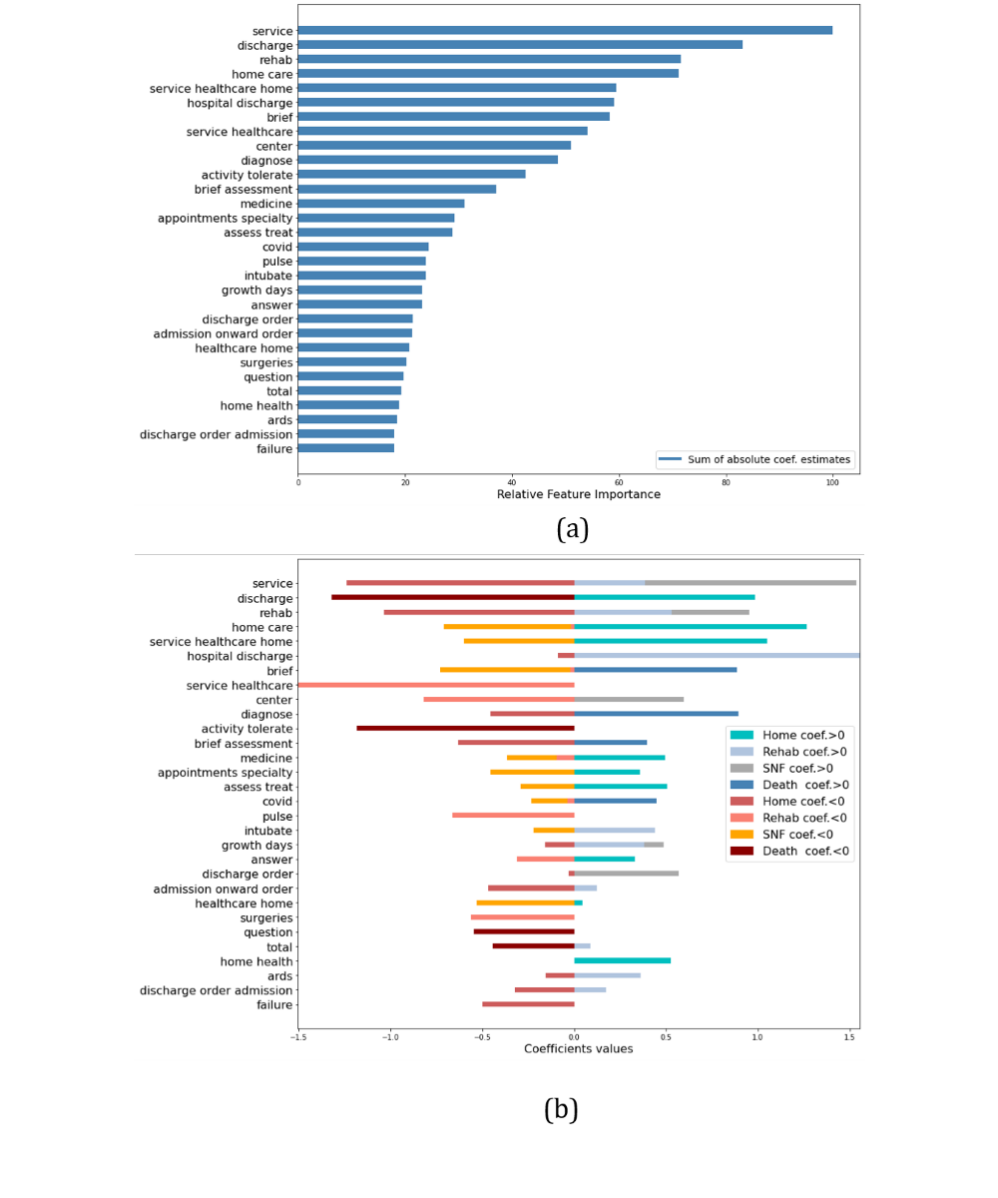
Relative importance of top 30 features obtained with the model coefficients estimates for (A) the sum of the absolute coefficients values and (B) the coefficients values discriminated by outcome. Coef: coefficient.

The best model configuration parameters and performance results in the hold-out test set are presented in Table 3 with 95% CIs. The corresponding confusion matrices normalized by precision and recall are presented in Figure 2. The performance discriminated by discharge outcome is presented in Table 4. Higher performance was obtained for the outcomes of home discharge and death compared to inpatient rehabilitation and SNIF discharge outcomes. The model presented higher recall (0.95) and precision (1.0) for the death outcome. Home disposition also presented high performance for these metrics. For this model, 2 deceased patients were classified as discharged home. In experiments, for models where we included the discharge disposition field, extracted from the discharge summary, all deceased patients were correctly classified. The inpatient rehabilitation outcome presented the lowest recall (0.61) and 12 patients with this outcome were incorrectly classified by the model as discharged to SNIF. The outcome of disposition to SNIF presented the lowest precision (0.68) overall and 20 patients discharged home were incorrectly predicted as discharged to SNIF. Compared to the initial set of features in the training vocabulary, the final model contained approximately 83% fewer features, with a total of 179 features. The relative importance of the top 30 model features is presented in Figure 3, where the importance for each feature consisted of the sum of the absolute coefficients’ values across the outcomes.

**Table 3:** Model performance in the hold-out test set and configuration parameters.

Area under the receiver operating characteristic curve^a^	Accuracy^a^	Recall^a^	F_1_ score^a^	Average precision^a^	Precision^a^	Parameters
0.98 (0.97-0.98)	0.88 (0.85-0.90)	0.88 (0.85-0.90)	0.88 (0.85-0.90)	0.81 (0.75-0.84)	0.88 (0.85-0.90)	Number of features (unigrams, bigrams, trigrams): 179 (95, 52, 32); C=0.09; warm start: true

^a^The 95% CIs of bootstrapping results are in parentheses.

**Table 4:** Model performance in the hold-out test set by discharge outcome.

Outcome	Area under the receiver operating characteristic curve^a^	Accuracy^a^	Recall^a^	F_1_ score^a^	Average precision^a^	Precision^a^
Home	0.97 (0.95-0.98)	0.92 (0.89-0.94)	0.92 (0.89-0.95)	0.93 (0.91-0.95)	0.92 (0.88-0.94)	0.94 (0.91-0.97)
Rehab	0.95 (0.91-0.98)	0.95 (0.93-0.97)	0.61 (0.53-0.76)	0.67 (0.53-0.78)	0.48 (0.32-0.64)	0.73 (0.58-0.86)
Skilled nursing inpatient facility	0.93 (0.88-0.96)	0.90 (0.87-0.92)	0.81 (0.72-0.88)	0.74 (0.64-0.79)	0.58 (0.46-0.66)	0.68 (0.58-0.75)
Death	1.00 (1.00-1.00)	0.99 (0.99-1.00)	0.95 (0.90-0.98)	0.97 (0.95-0.99)	0.95 (0.91-0.98)	1.00 (1.00-1.00)

^a^The 95% CIs of bootstrapping results are in parentheses.

“Service” was the feature assigned the highest importance for classification of the discharge outcomes. For inpatient rehabilitation and SNIF dispositions, the coefficient values for this feature are positive, which indicates that this term will most likely appear in the preprocessed notes for both outcomes. “Home care,” “healthcare home,” and “home health” were assigned a positive coefficient value for home disposition. “Service healthcare home” was also assigned high importance for this outcome, suggesting that this feature is related to patients discharged home with home health care services provided. “Medicine” and “appointments specialty” were also important for this outcome. “Rehab” had positive coefficients for both inpatient rehabilitation and SNIF dispositions. “Intubate” and “ARDS” (acute respiratory distress syndrome) are important features for inpatient rehabilitation disposition. For death, “discharge” and “activity tolerate” presented negative coefficient values, indicating that these features are unlikely to appear in discharge summaries of deceased patients. “Brief assessment” and “brief” are assigned high coefficient values for this outcome. “Covid” was assigned a positive coefficient value for predicting death, while the term was given negative values for inpatient rehabilitation and SNIF.

Training performance is depicted in Figure S1 in Multimedia Appendix 2, with the curve corresponding to the *R*^2^ scores for the different values of the inversed regularization strength. The top 15 features and their relative importance obtained with LASSO regularization are presented for each outcome (Figure S2 in Multimedia Appendix 2). Blue bars correspond to features with positive coefficient values and red bars to features with negative coefficient values. The areas under the ROC and Precision-Recall curves for the best model are also presented (Figure S3 in Multimedia Appendix 2). We also assessed how the model performance and the features selected as the most important in the train set varied with the dimension of the train set (Figure S4 in Multimedia Appendix 2). The hold-out test set for model evaluation was fixed and the train set dimension was varied from 10% to 100% of the original train set, with 1215 patients. We observed that the best performance was achieved with a higher number of patients in the train set (ie, the original train set of 100%). However, with 50% versus 100% of the original train set, the model achieved good performance for 1018 (versus 1056) vocabulary features (AUROC 0.97 versus 0.98 and AP 0.79 versus 0.81, respectively). We assessed the common features between each train set and the original train set (Figure S5 in Multimedia Appendix 2). Among the top 30 features, there were 10 common features between the 50% and the original train sets. A higher number of common features was found for the train set with 90% of the original train set, with a total of 17 common features. Finally, we observed that more than half of the features in the top 30 from the original train set were selected as top 30 in at least two train sets (Figure S6 in Multimedia Appendix 2).

## Discussion

### Principal Findings

In this study, a machine learning–based NLP pipeline was developed to classify the discharge disposition of adult patients hospitalized with COVID-19. The model achieved near-perfect identification of patients with outcomes of home disposition or death. For the intermediate outcomes of inpatient rehabilitation or SNIF, performance was imperfect but also acceptable. Due to this classification task being relatively easy, more complex and time-consuming modeling approaches, such as recurrent neural networks or bidirectional encoder representations from transformers were not considered. We acknowledge that for harder tasks, these approaches can improve performance. The final method is automated, thus enabling large-scale rapid processing of thousands of discharge summaries, a task that is infeasible when relying on manual chart review.

### Limitations

The present analysis was limited to a cohort of patients with COVID-19, who may have specific medical symptoms related to the disease. Therefore, as future work, it is proposed to extend the model to other cohorts. Further, although results spanned 2 hospitals, they are located in the same geographic region (Boston, Massachusetts). Thus, our cohort may not be representative of other US and non-US populations. Moreover, decision making for discharge disposition may vary for different hospitals, according to the number of SNIFs or rehabilitation centers in the geographic area, which may affect the generalizability of the model. The models were developed with textual information from discharge summaries, while the addition of other clinical features (eg, physical or occupational therapy reports, social work or case manager notes) was not considered, which is a limitation of the study and can be pursued in future work.

### Comparison With Prior Work

Extraction of information from clinical narratives is a growing application of NLP in health care. NLP has been used to extract information from hospital discharge notes about medical conditions such as postsurgical sepsis [21], pneumonia [22], or other potential medical problems [23], as well as to identify critical illness [24,25], detect adverse events [26], predict risk of rehospitalization [27], extract medication information [28], and risk stratify patients [29]. To the best of our knowledge, ours is the first work on classifying hospital discharge disposition based on discharge summary notes using machine learning and NLP.

### Conclusions

This study shows that a supervised learning–based NLP approach can be used to accurately classify the discharge disposition of hospitalized patients with COVID-19 in an automated fashion. This model, and the NLP approach used to develop it, have the potential to accelerate and increase the scale of research that is possible with EHR data.

## Appendix

Multimedia Appendix 1 Methodology.

Multimedia Appendix 2 Results.

## Abbreviations

AP: average precision
ARDS: acute respiratory distress syndrome
AUPRC: area under the precision recall curve
AUROC: area under the receiver operating characteristic curve
BoW: bag-of-words
BWH: Brigham and Women’s Hospital
EHR: electronic health record
FPR: false positive rate
ICU: intensive care unit
LASSO: least absolute shrinkage and selection operator
MGH: Massachusetts General Hospital
NLP: natural language processing
ROC: receiver operating characteristic
SNIF: skilled nursing inpatient facility

## References

[1] Richardson S, Hirsch JS, Narasimhan M, Crawford JM, McGinn T, Davidson KW, Barnaby DP, Becker LB, Chelico JD, Cohen SL, Cookingham J, Coppa K, Diefenbach MA, Dominello AJ, Duer-Hefele J, Falzon L, Gitlin J, Hajizadeh N, Harvin TG, Hirschwerk DA, Kim EJ, Kozel ZM, Marrast LM, Mogavero JN, Osorio GA, Qiu M, Zanos TP, the Northwell COVID-19 Research Consortium (2020). Presenting Characteristics, Comorbidities, and Outcomes Among 5700 Patients Hospitalized With COVID-19 in the New York City Area. JAMA.

[2] Holshue ML, DeBolt C, Lindquist S, Lofy KH, Wiesman J, Bruce H, Spitters C, Ericson K, Wilkerson S, Tural A, Diaz G, Cohn A, Fox L, Patel A, Gerber SI, Kim L, Tong S, Lu X, Lindstrom S, Pallansch MA, Weldon WC, Biggs HM, Uyeki TM, Pillai SK (2020). First Case of 2019 Novel Coronavirus in the United States. N Engl J Med.

[3] Fauci A, Lane H, Redfield R (2020). Covid-19 - Navigating the Uncharted. N Engl J Med.

[4] Zhou F, Yu T, Du R, Fan G, Liu Y, Liu Z, Xiang J, Wang Y, Song B, Gu X, Guan L, Wei Y, Li H, Wu X, Xu J, Tu S, Zhang Y, Chen H, Cao B (2020). Clinical course and risk factors for mortality of adult inpatients with COVID-19 in Wuhan, China: a retrospective cohort study. The Lancet.

[5] Wu Z, McGoogan JM (2020). Characteristics of and Important Lessons From the Coronavirus Disease 2019 (COVID-19) Outbreak in China: Summary of a Report of 72 314 Cases From the Chinese Center for Disease Control and Prevention. JAMA.

[6] CDC COVID-19 Response Team (2020). Severe Outcomes Among Patients with Coronavirus Disease 2019 (COVID-19) - United States, February 12-March 16, 2020. MMWR Morb Mortal Wkly Rep.

[7] He X, Lau EHY, Wu P, Deng X, Wang J, Hao X, Lau YC, Wong JY, Guan Y, Tan X, Mo X, Chen Y, Liao B, Chen W, Hu F, Zhang Q, Zhong M, Wu Y, Zhao L, Zhang F, Cowling BJ, Li F, Leung GM (2020). Temporal dynamics in viral shedding and transmissibility of COVID-19. Nat Med.

[8] Lauer SA, Grantz KH, Bi Q, Jones FK, Zheng Q, Meredith HR, Azman AS, Reich NG, Lessler J (2020). The Incubation Period of Coronavirus Disease 2019 (COVID-19) From Publicly Reported Confirmed Cases: Estimation and Application. Ann Intern Med.

[9] Wang D, Hu B, Hu C, Zhu F, Liu X, Zhang J, Wang B, Xiang H, Cheng Z, Xiong Y, Zhao Y, Li Y, Wang X, Peng Z (2020). Clinical Characteristics of 138 Hospitalized Patients With 2019 Novel Coronavirus-Infected Pneumonia in Wuhan, China. JAMA.

[10] The Covid-19 Tracker. STAT.

[11] Nallapati R, Zhou B, Nogueira dos santos C, Gulcehre C, Xiang B Abstractive text summarization using sequence-to-sequence RNNs and beyond. ArXiv.

[12] Hirschberg J, Manning CD (2015). Advances in natural language processing. Science.

[13] Wilbur WJ, Rzhetsky A, Shatkay H (2006). New directions in biomedical text annotation: definitions, guidelines and corpus construction. BMC Bioinformatics.

[14] Buchan NS, Rajpal DK, Webster Y, Alatorre C, Gudivada RC, Zheng C, Sanseau P, Koehler J (2011). The role of translational bioinformatics in drug discovery. Drug Discov Today.

[15] Nadkarni PM, Ohno-Machado L, Chapman WW (2011). Natural language processing: an introduction. J Am Med Inform Assoc.

[16] Uzuner Ö, South B, Shen S, DuVall S (2011). 2010 i2b2/VA challenge on concepts, assertions, and relations in clinical text. J Am Med Inform Assoc.

[17] Tibshirani R (2018). Regression Shrinkage and Selection Via the Lasso. Journal of the Royal Statistical Society: Series B (Methodological).

[18] Cramer JS (2003). The Origins of Logistic Regression. SSRN Journal.

[19] Bühlmann P, van de Geer S (2011). Statistics for High-Dimensional Data: Methods, Theory and Applications.

[20] Azari A, Janeja V, Levin S (2015). Imbalanced learning to predict long stay Emergency Department patients.

[21] Arvind V, Cho B, Ukogu CO, Kim J, Cho SK (2018). Wednesday, September 26, 2018 2:00 PM – 3:00 PM Integrating Technology into Practice: 59. Natural language processing of electronic medical records can identify sepsis following orthopedic surgery. The Spine Journal.

[22] Yetisgen-Yildiz M, Glavan B, Xia F, Vanderwende L, Wurfel M (2011). Identifying patients with pneumonia from free-text intensive care unit reports. https://faculty.washington.edu/melihay/publications/ICML_2011.pdf.

[23] Meystre S, Haug P (2006). Improving the sensitivity of the problem list in an intensive care unit by using natural language processing. AMIA Annu Symp Proc.

[24] Weissman GE, Harhay MO, Lugo RM, Fuchs BD, Halpern SD, Mikkelsen ME (2016). Natural Language Processing to Assess Documentation of Features of Critical Illness in Discharge Documents of Acute Respiratory Distress Syndrome Survivors. Annals ATS.

[25] Marafino BJ, Park M, Davies JM, Thombley R, Luft HS, Sing DC, Kazi DS, DeJong C, Boscardin WJ, Dean ML, Dudley RA (2018). Validation of Prediction Models for Critical Care Outcomes Using Natural Language Processing of Electronic Health Record Data. JAMA Netw Open.

[26] Murff HJ, Forster AJ, Peterson JF, Fiskio JM, Heiman HL, Bates DW (2003). Electronically screening discharge summaries for adverse medical events. J Am Med Inform Assoc.

[27] Kang Y, Hurdle J (2020). Predictive Model for Risk of 30-Day Rehospitalization Using a Natural Language Processing/Machine Learning Approach Among Medicare Patients with Heart Failure. Journal of Cardiac Failure.

[28] Yang H (2010). Automatic extraction of medication information from medical discharge summaries. J Am Med Inform Assoc.

[29] Lehman L, Saeed M, Long W, Lee J, Mark R (2012). Risk stratification of ICU patients using topic models inferred from unstructured progress notes. AMIA Annu Symp Proc.

