# Supplementary material for: Classification of the Disposition of Patients Hospitalized with COVID-19: Reading Discharge Summaries Using Natural Language Processing
Source: JMIR Med Inform. 2021 Feb 10;9(2):e25457. doi: 10.2196/25457 (PMC7879729; doi:10.2196/25457)
Supplement: Multimedia Appendix 1 [file medinform_v9i2e25457_app1.doc]

## Multimedia Appendix 1

Table S1. List of abbreviations expansion and spell corrections from the lemmatization in the notes preprocessing stage.

| **Abbreviation, expansion** |
| --- |
|  |
| 'abd', 'abdominal'  ‘abdo','abdominal'  'afib', 'atrial fibrillation'  'covi', 'covid'  'diagno', 'diagnosis'  'orient', 'orientation'  'tota', 'total'  'disch', 'discharge'  'demonst', 'demonstrate'  'servi', 'services'  'requir', 'require'  'preferen', 'preference'  'preferenc', 'preference'  ‘caregive', ‘caregiver' |

Table S2. List of stopwords removed from the notes in the preprocessing stage.

| **Stopwords**  'i', 'me', 'my', 'myself', 'we', 'our', 'ours', 'ourselves', 'you', ‘youre’, ‘youve', ‘youll’, ‘youd’, 'your', 'yours', 'yourself', 'yourselves', 'he', 'him', 'his', 'himself', 'she', "shes", 'her', 'hers', 'herself', 'it', 'its', 'itself', 'they', 'them', 'their', 'theirs', 'themselves', 'the', 'with', 'to', 'be', 'from', 'which', 'dob', 'date', 'summary', 'please', 'dear', 'fa', 'facesheet', 'wi', 'llst', 'items', 'st', 'same', 'phone', 'sign', 'about', 'should', 'as', 'or', 'an', 'for', 'of', 'ac', 'in', 'by', 'at', 'fo', 'me', 'nan', 'hea', 'pro', 'and', 'up', 'full', 'code', 'have', 'has', 'is', 'part', 'do', 'will', 'there', 'faci', 'this', 'that', 'what', 'nc', 'comment', 'other', 'throughout', 'md', 'mdd', 'qd', 'per', 'sig', 'bid', 'when', 'use', 'while', 'apt', 'resu', 'con', 'dis', 'go', 'doct', 'mch', 'wnl', 'ml', 'mg', 'diff', 'tid', 'id', 'hs', 'medic', 'contact', 'but', 'hid', 'post', 'nt', 'first', ‘if', 'then', ‘who’, ‘whom’, ‘these’, ‘those’, ‘am’, ‘are’, ‘was’, ’were’, ‘been’, ‘being’, 'had', 'having', 'does', 'did', 'doing', 'because', 'until', 'against', 'between', 'into', 'through', 'during', 'before', 'after', 'above', 'below', 'down', 'out', 'on', 'off', 'over', 'under', 'again', 'further', 'once', 'here', 'when', 'where', 'why', 'how', 'all', 'any', 'both', 'each', 'few', 'more', 'most', 'some', 'such', 'nor', 'only', 'own', 'so', 'than', 'too', 'very', 'can', 'just', 'don', 'now' |
| --- |

Table S3. Example of discharge summary notes for a patient where protected health information (PHI) was removed, including the changing of dates.

| **Discharge summary notes** |
| --- |
| Physician Discharge Summary Admit date: Discharge date: Patient Information y.o. (DOB = //) Home Address: Home Phone: (home) What language do you prefer to use when discussing your healthcare?: English What language do you prefer for written communication?: English Type of Advance Care Directive(s): None Does patient have a Health Care Proxy form completed?: Patient declines Health Care Agents There are no Health Care Agents on file. Code Status at Discharge: Full Code (Confirmed) Hospitalization Summary Reason for Admission: Fever, Cough, COVID-19 Exposure Principal Problem: 2019 Novel Coronavirus Disease (Covid-19) Resolved Problems: * No resolved hospital problems. * Surgical (OR) Procedures: Surgeries this admission None Procedures this admission None Non (OR) Procedures: Items for Post-Hospitalization Follow-Up: PCP [ ] Please follow up COVID-19 results and resolution of respiratory symptoms. Pending Results Procedure Component Value Ref Range Date/Time COVID-19 RT PCR [] Collected: // Lab Status: In process Updated: // Hospital Course with depression, OSA on CPAP, who has a wife positive for COVID19, presents with cough and fever c/f COVID19 a/f monitoring ----- HPI ----- with depression, OSA on CPAP, presents with progressive cough and fever, and confusion that prompted the wife (cell) to call 911. Per EMS report, patient was back to normal baseline mental status. Patient's wife was transported in the ambulance and since she is know COVID-19 positive, patient was sent home with the ambulance.   On both husband and wife presented to the emergency department last week with body aches, myalgia, feeling unwell -Wife tested positive for corona virus, husband tested negative. Husband said he had started to feel "off" from his baseline   Approximately 3 days prior to admission, patient started having fevers -Fevers have been coming down with Tylenol and ibuprofen although the fever curve is rising -Fevers to 101, 102 °F -This morning had fever to 101.7 - Upon waking from sleep, was a little bit more confused than normal - Went to the bathroom, was feeling hot, took temperature and found it was 101.7 - Was not wearing CPAP overnight as usually does - patient was found to be confused by wife - wife reported patient has been sleeping longer than usually   Upon arrival to the ED, patient was satting well on RA, in no distress. He became febrile to 100.5F, otherwise vitals have been stable. He was given Tylenol and tessalon pearls. Labs s/f negative procal, normal WBC, and negative respiratory viral panel. SARS-COV2 swab was sent and is pending. CXR showed "Equivocal faint hazy upper lung opacities bilaterally". Upon my interview, patient said he was feeling fine. +dry cough, congestion, poor appetite. He had some diarrhea earlier the day prior which he thought was from ibuprofen but hadn't had any since. No SOB, CP, N/V, HA, dizziness. ----- SPU Course ----- #C/f COVID19 #Fever, cough Patient presents with 3 days of symptoms. Fever, fatigue, dry cough, anorexia. His wife is positive for COVID19. Differential diagnosis includes COVID vs other viral illness. Forunately, no leukopenia of LFT abnormalities. Has negative flu/ RSV/ Adeno/ Human metapneumovirus PCR/ Rhinovirus/ Parainfluenza/ Influenza A, B, RSV negative. Not likely to be bacterial, negative procal (0.10). Respiratory status stable. Patient had one episode of fever to 100.5 F during admission but defervesced with Tylenol and has been afebrile since. Very low risk for decompensation given age and no medical comorbidities. Patient was admitted for COVID-19 rule out given episode of confusion at home and CXR finding of equivocal faint hazy upper long opacities. Repeat CXR was stable. Patient's cough was managed with tessalon and robitussin PRN. At the time of discharge patient's COVID-19 RT PCR was pending. Given clinical stability, patient was discharged to home with instructions for home isolation. Boston Department of Health was called and informed of patient's case. Patient was instructed to self isolate and given strict instructions regarding return precautions. #Depression C/h wellbutrin 300mg daily Medications Allergies: Patient has no known allergies. Prior to Admission Medications Prescriptions buPROPion (WELLBUTRIN XL) 300 MG ER 24 hr tablet Sig: Take 300 mg by mouth daily. Facility-Administered Medications: None Medication List TAKE these medications Instructions benzonatate 100 MG capsule Commonly known as: TESSALON Last time this was given: Take 1 capsule (100 mg total) by mouth 3 (three) times a day as needed for cough. buPROPion 300 MG ER 24 hr tablet Commonly known as: WELLBUTRIN XL Take 300 mg by mouth daily. ondansetron 4 MG tablet Commonly known as: ZOFRAN Take 1 tablet (4 mg total) by mouth every 12 (twelve) hours as needed for nausea. Where to Get Your Medications These medications were sent to CVS/pharmacy Phone benzonatate 100 MG capsule ondansetron 4 MG tablet Hospital Care Team Service: Medicine Inpatient Attending: Attending phys phone: Discharge Unit: Primary Care Physician: Not Required Pcp None Transitional Plan Scheduled appointments: Signed Discharge Orders (From admission, onward) Ordered // Activity as tolerated // Discharge diet Comments: Diet Regular // For immediate questions regarding your hospitalization, your medications, and any pending test results please contact your doctor in the hospital: MD. Comments: For immediate questions regarding your hospitalization, your medications, and any pending test results please contact your doctor in the hospital: MD. Discharge instructions and important events and results You were admitted to BWH on // with cough and fever. Given your recent exposure to COVID-19, you were admitted to the Special Pathogens Service and tested for this virus. These results were still tested at the time of your discharge. Given the stability in your respiratory symptoms, you were discharged with a plan to self-quarantine at home until your results return. You will be contacted regarding the results by your local department of health and the best next steps after receiving these results. You can take Tylenol 650 mg every 8 hours to treat your fevers. Your last dose of 975 mg was at 12:40 PM today. You can start taking Tylenol 650 mg every 8 hours starting at 6 PM this evening. You have been discharged home with new medications: - Tessalon Perles 100 mg three times daily as needed for cough. - Zofran 4 mg twice daily as needed for nausea. Please contact us with any questions or concerns. It was a pleasure taking care of you and we wish you a speedy recovery! Sincerely, The BWH team Exam Temperature: 37.7 °C (99.8 °F) (03/08/20 1532) | Heart Rate: 95 (03/08/20 1532) | BP: 121/77 (03/08/20 1532) | Respiratory Rate: 16 (03/08/20 1225) | SpO2: 100 % (03/08/20 1532) O2 Device: None (Room air) (03/08/20 1532) | Weight: 97.5 kg (215 lb) (03/08/20 2315) Height: 172.7 cm (5' 8") (03/07/20 2315) BMI (Calculated): 32.7 (03/07/20 2315) Discharge Exam Significant Discharge Exam Findings: General: Well-developed, no apparent distress HEENT: PERRL, EOMI, moist mucous membranes Cardiovascular: Regular rate, regular rhythm Pulmonary: Clear to auscultation bilaterally Abdomen: Soft, nontender, nondistended. No rebound tenderness. MSK: Moves extremities spontaneously Skin: No lesions on chest, face, upper extremities. Neuro: No gross focal neuro deficits Psych: Appropriate affect, thought content normal.    Orientation Level: Oriented X7 Cognition: Follows commands Speech: Clear Vision: Functional Hearing: Functional Assistive Devices: None Data/Results Results are shown for the following tests if performed (CBC, Chem 7, Mg, Coag). If the patient did not have any of these tests, no results will be shown here. Lab Results Component Value Date/Time WBC 4.57 // 0545 RBC 5.35 // 0545 HGB 14.8 //0545 HCT 45.5 // 0545 MCH 27.7 //0545 MCV 85.0 //0545 PLT 157 //0545 RDW 12.7 // 0545 Lab Results Component Value Date/Time NA 138 //0545 K 3.6 // 0545 CL 98 // 0545 CO2 25 // 0545 BUN 7 // 0545 CRE 1.18 // 0545 CA 8.4 (L) // 0545 GLU 122 (H) // 0545 |

Table S4. Performance metrics used for model assessment.

| **Performance metrics**  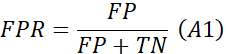  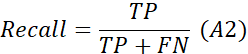  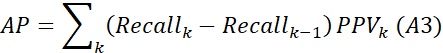  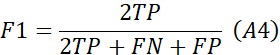  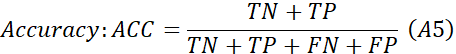  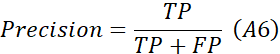  TN and TP indicate the true negatives and true positives; FN and FP indicate the false negatives and false positives.  For the multiclass problem, for each class *c*:  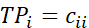  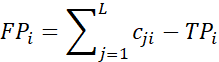  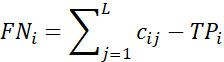  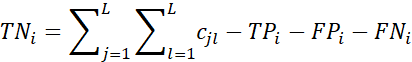  where each element 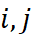 corresponds to the number of items with true class *i* that were classified as being in class *j,* with 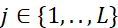 and *L* as the total number of classes. |
| --- |
