# Supplementary material for: Classification of the Disposition of Patients Hospitalized with COVID-19: Reading Discharge Summaries Using Natural Language Processing
Source: JMIR Med Inform. 2021 Feb 10;9(2):e25457. doi: 10.2196/25457 (PMC7879729; doi:10.2196/25457)

## Multimedia Appendix 2

Figure S1. Model average training performance in 5-fold cross validation for different values of the inversed regularization strength constant C.


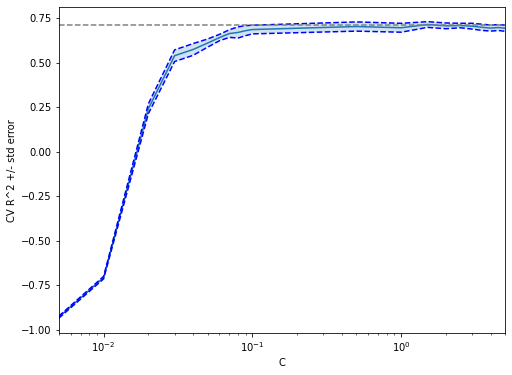


Figure S2. Relative importance of top 15 features obtained for the binary “one-vs-rest” model for each discharge outcome: (a) home; (b) inpatient rehabilitation; (c) skilled inpatient nursing facility; and (d) death.


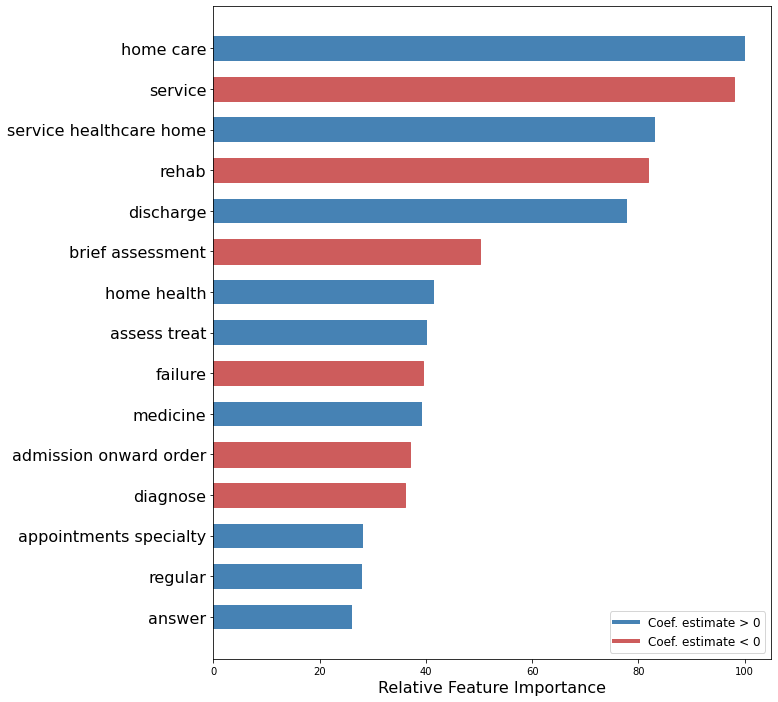

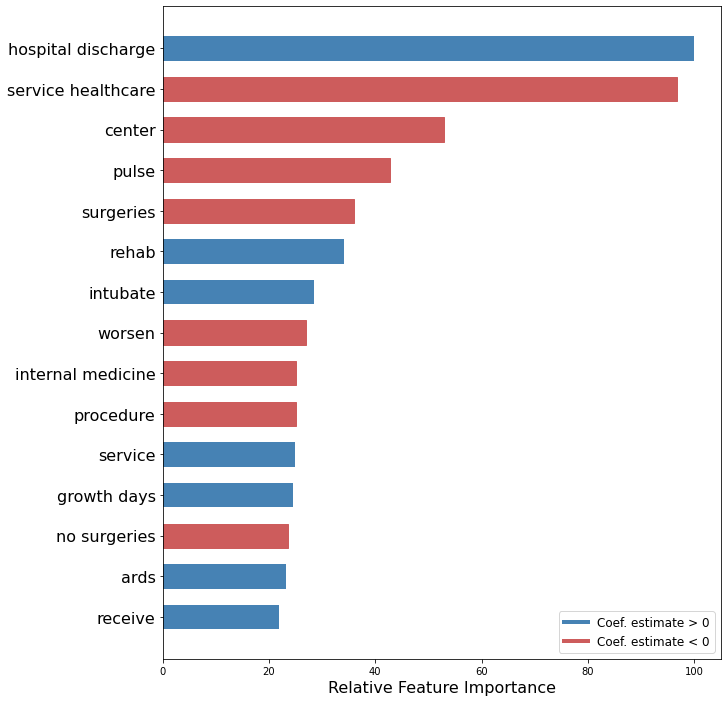


(a)(b)


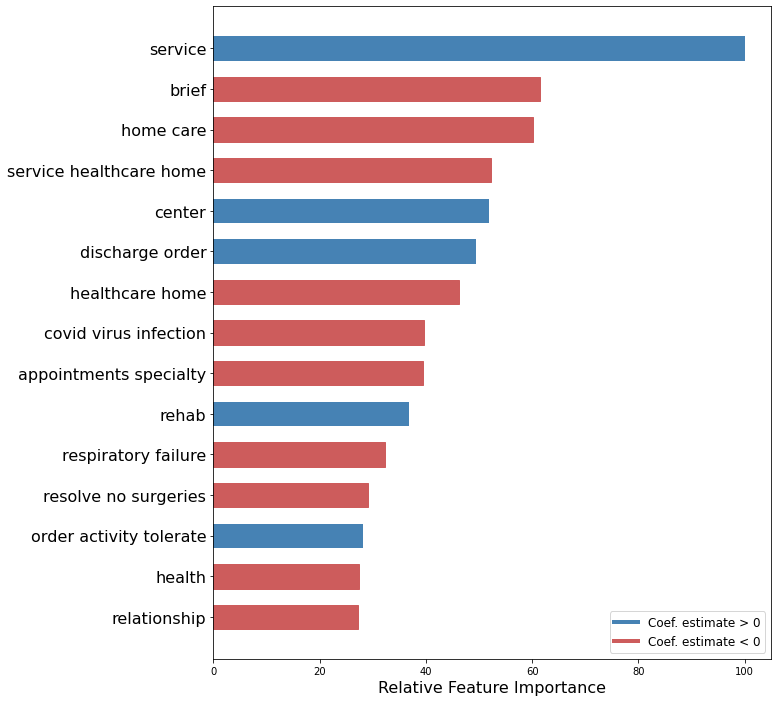

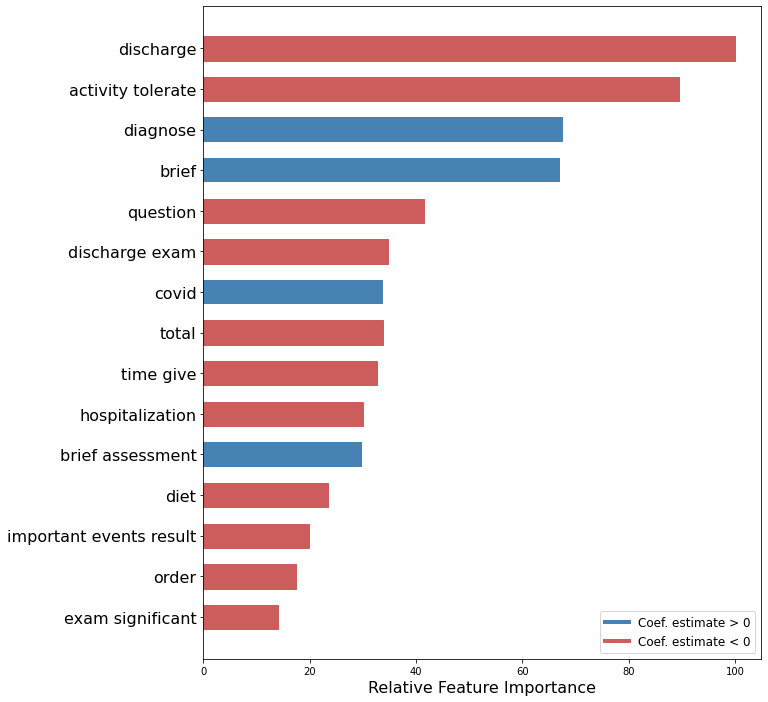


(c) (d)

Figure S3. Areas under the (a) ROC curve (AUROC), and (b) Precision-Recall curve (AUPRC), for the best model evaluated in the hold out test set.


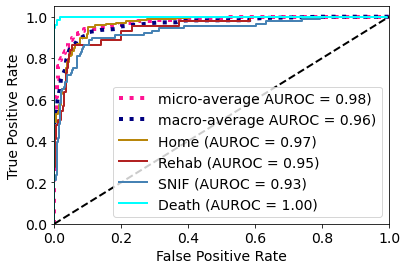


(a)


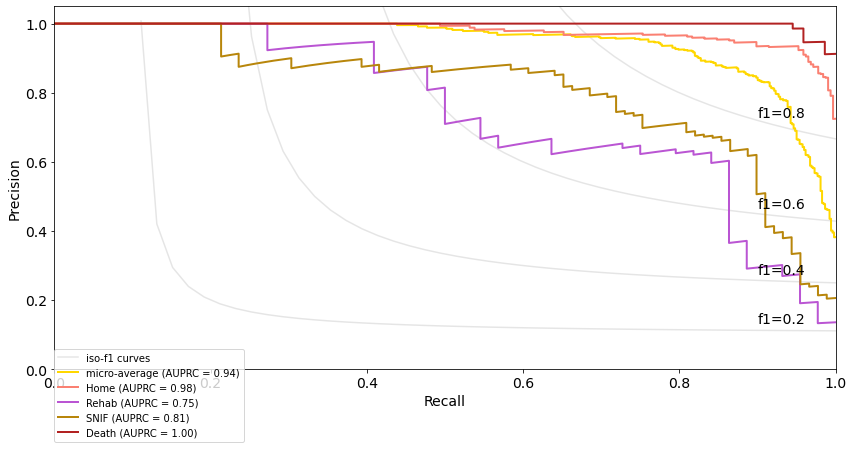


(b)

Figure S4. (a) Model performance for the best model evaluated in the hold out test set and (b) number of selected features in train, according to each train set (dimensions 10-100% of the original train set).


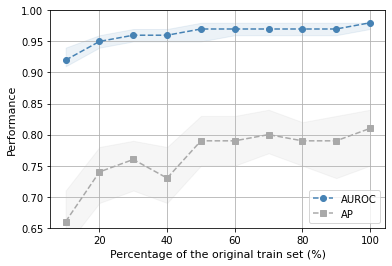

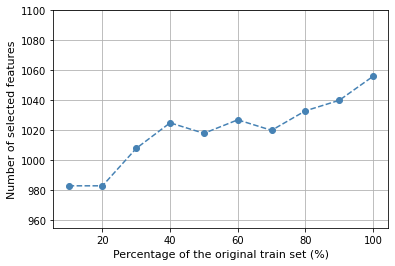


1. (b)

Figure S5. Number of common features between the top 30 features selected for each train set (dimensions 10-100% of the original train set) and the top 30 features from the original train set.


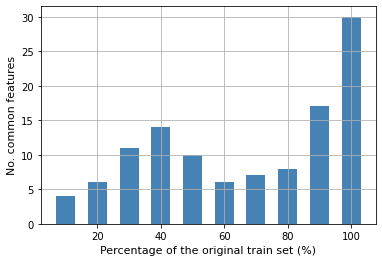


Figure S6. Frequency of features selected, among all train sets (dimensions 10-100% of the original train set), for the top 30 features selected from the original train set.


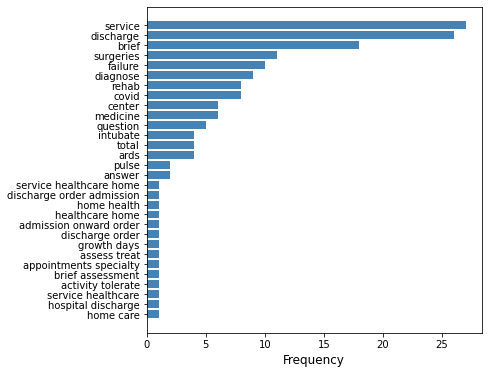

Supplement: Multimedia Appendix 2 [file medinform_v9i2e25457_app2.doc]
